# Mechanisms Promoting the Long-Term Persistence of a *Wolbachia* Infection in a Laboratory-Adapted Population of *Drosophila melanogaster*

**DOI:** 10.1371/journal.pone.0016448

**Published:** 2011-01-24

**Authors:** Urban Friberg, Paige M. Miller, Andrew D. Stewart, William R. Rice

**Affiliations:** 1 Department of Evolutionary Biology, Uppsala University, Uppsala, Sweden; 2 Department of Ecology, Evolution and Marine Biology, University of California Santa Barbara, Santa Barbara, California, United States of America; Field Museum of Natural History, United States

## Abstract

Intracellular bacteria of the genus *Wolbachia* are widespread endosymbionts across diverse insect taxa. Despite this prevalence, our understanding of how *Wolbachia* persists within populations is not well understood. Cytoplasmic incompatibility (CI) appears to be an important phenotype maintaining *Wolbachia* in many insects, but it is believed to be too weak to maintain *Wolbachia* in *Drosophila melanogaster*, suggesting that *Wolbachia* must also have other effects on this species. Here we estimate the net selective effect of *Wolbachia* on its host in a laboratory-adapted population of *D. melanogaster*, to determine the mechanisms leading to its persistence in the laboratory environment. We found *i*) no significant effects of *Wolbachia* infection on female egg-to-adult survival or adult fitness, *ii*) no reduced juvenile survival in males, *iii*) substantial levels of CI, and *iv*) a vertical transmission rate of *Wolbachia* higher than 99%. The fitness of cured females was, however, severely reduced (a decline of 37%) due to CI in offspring. Taken together these findings indicate that *Wolbachia* is maintained in our laboratory environment due to a combination of a nearly perfect transmission rate and substantial CI. Our results show that there would be strong selection against females losing their infection and producing progeny free from *Wolbachia*.

## Introduction

*Wolbachia* is an intracellular bacterium that is widespread among arthropod and nematode taxa [1], [2], [3], [4], [5]. It is effectively matrilineally propagated because an infected female's sons and daughters are both infected with *Wolbachia* at high frequency, but the sons do not transmit it to their offspring. This matrilineal mode of transmission causes selection to favor *Wolbachia* variants that manipulate the reproductive biology of its hosts, to better serve the transmission of the bacterium (reviewed in [6]). Several different phenotypes produced by *Wolbachia* have been reported that promote its fitness; parthenogenesis of its host, male-killing, and cytoplasmic incompatibility (CI) (reviewed in [7]). These phenotypes commonly reduce the fitness of the host's nuclear genome, but *Wolbachia* has also been found to help its host both in terms of increased resistance to viral infections (e.g. [8]) and iron homeostasis [9]. As a result, *Wolbachia* is predicted to be an influential selective agent in its host insect's evolution [10]. An understanding of how *Wolbachia* persists in populations is therefore critical for our understanding of evolution in a wide diversity of taxa.

Several past studies have documented how *Wolbachia* is maintained in natural populations [11], [12], [13]. These studies focused primarily on a single population of *D. simulans* and all of the relevant parameter values required to fully evaluate how *Wolbachia* persists were estimated. With respect to infection rates, these parameter estimates, when integrated into theoretical models [14], [15], [16], conform surprisingly well with the patterns of infection found in nature [17]. This close correspondence between observed and expected values suggests that the mechanism by which *Wolbachia* is maintained is largely understood, at least in one natural population.

The maintenance of *Wolbachia* in *D. melanogaster* is less well understood. Recent evidence indicates that a new *Wolbachia* strain has rapidly spread over the world within the last 70 years [18]. This is particularly puzzling since *Wolbachia* in this species often displays only a weak, or no, CI phenotype [19], [20], [21], [22], [23], although it may be more pronounced in young males [12], [23], [24]. One solution to this discrepancy is the hypothesis that *Wolbachia* has a positive influence on fitness of female *D. melanogaster* that compensates for its weak CI [21], [25], [26], but current evidence is mixed. While most studies find no influence of *Wolbachia* on female fitness [21], [25], [27], [28], some studies do report a positive effect [29], [30], while others report a negative effect [29], [30]. It should be noted, however, that most of these measures were restricted to fitness components and did not encompass a complete estimate of female fitness.

*Wolbachia* is widespread among wild [26] and laboratory populations of *D. melanogaster* [31], and serves as a model organism for both evolutionary and molecular biology. Understanding *Wolbachia's* role in this species is therefore important. Here we take advantage of a large outbred population of *D. melanogaster* (LH_M_) that has adapted to a competitive laboratory environment for over 400 generations [32], [33]. This population reproduces semelparously and has a two-week non-overlapping life history. Because of these characteristics, it is possible to measure lifetime fitness for large numbers of individuals and to break down this metric into its juvenile and adult components. These characteristics make it feasible to obtain the full set of measurements needed to determine the mechanisms responsible for maintaining *Wolbachia* infection. Importantly, all of these measurements can be made in the environment to which the flies and *Wolbachia* have adapted for many hundreds of generations. Our results show that *Wolbachia* has surprisingly few fitness effects in the LH_M_ population, and that it is maintained due to a combination of substantial CI coupled with a high vertical transmission rate.

## Methods

### Fly stocks

All flies used in this experiment were derived from LH_M_, a large (∼10,000 eggs at the start of each generation and about 1,800 breeding adults), outbred population that had adapted to a constant laboratory environment and culturing regime for more than 400 generations when this study was initiated. LH_M_ is cultured on a 14-day discrete generation cycle at 25°C on a 12-h∶12-h light∶dark cycle. The culture cycle is started at day 0 with 56 ‘juvenile competition vials’ (37-ml vials containing 6 ml of cornmeal-molasses-killed-yeast-medium), each containing 150–200 eggs. In these vials, juveniles compete over resources, pupate and spend their early adult stages. At day 11.25, a randomized sample of 1792 adults from these vials are transferred, in groups of 16 pairs, to 56 ‘adult competition vials’ with a prescribed amount (3.84 mg) of live yeast on top of the culture medium. In these vials, males compete for fertilization opportunities and females compete for a limited supply of live yeast (the amount of yeast consumed is proportional to female fitness [34]). At day 13.25, the adult flies are flipped into fresh vials where eggs are laid over an 18-hour period, completing the 14-day lifecycle. This culturing regime makes it feasible to measure net fitness in this population. For a detailed description of this population and its culturing protocol see [32].

In addition to the LH_M_ base population, three populations derived from this population were used in our experiments: LH_M_-*bw*, LH_M_-W^-^ and LH_M_-*bw*-W^-^. LH_M_-*bw* had been established hundreds of generations prior to the start of these experiments by sequentially backcrossing the recessive eye-color marker *bw*, over nine generations, into LH_M_. To prevent divergence in genetic backgrounds, the LH_M_-*bw* population has been recurrently backcrossed to the LH_M_ population at approximately one year intervals. At the start of this study LH_M_ and LH_M_-*bw* were tested for the presence of *Wolbachia*, using PCR (see Snook et al. 2000), and both found to be positive (data not shown). To create *Wolbachia* cured replicas of LH_M_ and LH_M_-*bw*, a large sample of each of these populations was treated with tetracycline over two generations following the protocol of Hoffman et al. [24]. We subsequently confirmed that these new populations (LH_M_-W^-^ and LH_M_-*bw*-W^-^, W^-^ for *Wolbachia* negative) were cured from *Wolbachia* infection, again using PCR (data not shown).

### Backcrossing

Tetracycline treatment potentially has a selective effect [35]. To make sure no genetic differences existed between the LH_M_-W^-^ and LH_M_ populations, we backcrossed LH_M_-W^-^ to LH_M_ for seven generations. For each backcross we used 56 vials of 16 non-virgin 1–2-day-old LH_M_-W^-^ females and 16 5-day-old LH_M_ males (males of this older age class were used since CI has been found to decrease with male age [12]. Since we used non-virgin females, we simultaneously tested how efficient this backcrossing technique was by conducting two separate assays. In the first assay, we added 16 1–2-day-old non-virgin LH_M_-*bw*-W^-^ females to 16 5–6-day-old LH_M_ males (16–20 vials with 16 pairs generations 1–2, 4–7). In the second assay, we added 16 1–2-day-old non-virgin LH_M_-*bw* females to 16 5–6-day-old LH_M_ males (19–20 vials generations 4–7). The efficiency of the backcrossing was estimated by the proportion of red offspring sired in each vial (i.e. red offspring fathered by males from LH_M_ and brown from LH_M_-*bw* or LH_M_-*bw*-W^-^, depending on the cross). The rational for using LH_M_-*bw*-W^-^ as the female control population during the first two generations of backcrossing was that it also had experienced the tetracycline treatment. However, as the potential maternal effects from the tetracycline treatment waned over the first generations post tetracycline treatment, and as the genome of the LH_M_-W^-^ females gradually become more similar to that of the LH_M_ populations (assuming that the tetracycline treatment had a selective effect), we decided to test females from both LH_M_-*bw*-W^-^ and LH_M_-*bw* in the subsequent generations. The average proportions of the genome replaced per generation, for LH_M_-*bw*-W^-^ and LH_M_-*bw*, during generations 4–7 were very similar (45.6% and 46.4% respectively), and we therefore pooled the data from these two groups when estimating the rate of backcrossing. The overall backcrossing rate was estimated by bootstrapping the data. From each generation we randomly sampled 56 estimates with replacement (since there were 56 vials in the actual population that was backcrossed), and from this we calculated the percentage of the genome that was replaced over the 7 generations. We then repeated this procedure 10,000 times to estimate a confidence interval of the mean. Since we did not have data from generation 3 we created pseudo data for this generation by pooling the data from the second and fourth generation. This procedure estimated that 98.47% (98.39%, 98.54%, lower and upper confidence interval) of the genome of the LH_M_-W^-^ population was replaced with the LH_M_ genetic background, after seven generations.

### Cytoplasmic incompatibility

CI has been shown to decline with male age [12], [23], [24]. We therefore tested for CI with males that were 1 day old and males that were 3 days old. These two age classes cover a substantial part of the relevant male ages in this population, since most males are discarded when 4 days old (and males can never become older than 5 days), due to the discrete generation culturing protocol. Males of these two age classes, both from LH_M_ and LH_M_-W^-^, were mated to virgin LH_M_ and LH_M_-W^-^ 3-day-old females, in all four combinations. We tested all four combinations to rule out any indication that CI was not due to an inherent difference between the LH_M_ and LH_M_-W^-^ populations. To measure the hatch rate of eggs, 25 pairs of males and virgin females were combined in a vial and mass-mated for 7 hours. After the mating period, the males were discarded and the females were transferred to inverted half-pint containers. These containers were placed on small Petri dishes filled with fly medium and with a small amount of hydrated live yeast applied to the surface. After 15 hours of egg-laying the females were discarded and 200 eggs were counted out in piles of 20 on the food surface of each Petri dish. The number of unhatched eggs was then scored 27 hours later and re-scored after an additional 24 hours. Eggs were thus 27–43 hours when scored the first time and 51–67 hours when scored the second time. In total, 27 replicates per mating combination and male age were scored, divided over 3 independent blocks.

We initially attempted to analyze our data on hatchability assuming binominal error terms, but goodness of fit tests consistently indicated a lack of fit to this model, because of over-dispersion. We therefore used ANOVA and analyzed the mean values per replicate (each mean was the average of 9 data points). We used log transformed data to be able to compare changes in hatchability at different time points and at different sire ages. The model we used was *Y_ijkl_* = *μ* + *cross_i_* + *male age_j_* + *age of eggs_k_* + *cross_i_* × *male age_j_* + *cross_i_* × *age of eggs_k_* + *male age_j_* × *age of eggs_k_* + *cross_i_* × *male age_j_* × *age of eggs_k_* + *block_l_* + *e_ijkl_*, where block was treated as a random effect while all other factors were treated as fixed. Residual analysis confirmed the suitability of this statistical approach.

### Population infection status

We estimated the proportion of females infected by *Wolbachia* in the LH_M_ population by testing for the frequency of females that did not show reduced egg hatch rate (CI) when mated to infected males. We began by mass-mating 24 3-day-old LH_M_ females to 32 1-day-old males from either LH_M_ or LH_M_-W^-^, for 5 hours. We then put the females in individual vials and scored the proportion of females that showed elevated rates of egg inviability (low hatch rate = <80% hatch), 25–43 hours post egg-laying. In total, 1008 females from each cross were scored. In both types of crosses females could fail to produce hatching eggs either because they, or their mate, were unfertile. In addition, uninfected females crossed to LH_M_ males (infected) would also produce eggs with reduced hatch rate due to CI. Since several previous studies have shown that virtually all similarly aged virgin females mate within a 2 hour period ([36], [37], also in a recent unpublished assay of LH_M_, only 2 of 480 virgin females failed to mate during a 2 hour male exposure period), we concluded that differences in hatching of eggs between the groups of females would be unlikely to result from differential mating rates between the two groups. The estimated proportion of infected females also gave us an upper bound of the per generation loss of infection rate, including both leaky transmission and spontaneous cure rate. To reduce the influence of sampling error, we only included those females that produced at least 10 eggs in our statistical analysis.

### Adult fitness for infected and uninfected males and females

To test for an effect of *Wolbachia* on male and female fitness we conducted similar, but separate assays for each sex. To measure fitness of females, infected with or cured from *Wolbachia*, we deposited 60 eggs from either LH_M_ or LH_M_-W^-^ together with 120 eggs from LH_M_-*bw*-W^-^ into juvenile competition vials. After 11.25 days in these vials, 3 sets of 5 LH_M_ or 5 LH_M_ -W^-^ females were extracted from each vial and placed in an adult competition vial with 11 LH_M_-*bw*-W^-^ competitor females and 16 LH_M_-*bw*-W^-^ males (to ensure no CI). After two days of adult competition, the 5 target females (LH_M_ or LH_M_- W^-^) were transferred to individual test tubes for egg-laying. Eighteen hours later, the females were discarded and the number of eggs laid were counted and taken as a measure of female fitness. In total, 900 infected and 900 uninfected females were scored, across two independent blocks.

To create independent data points when analyzing female fitness, we took the average fecundity of the 15 females that initially had shared the same vial. These averages were then analyzed with ANOVA to test for differences in fitness between infected and uninfected females, using the following model: *Y_ijk_* = *μ + infection_status_i_* + *block_j_* + *e_ijk_*. Infection status was modeled as a fixed effect, while block was modeled as a random effect.

Male fitness was measured by rearing target and competitor males in the same vials and then measuring the reproductive success of the target males. We began by placing 60 target eggs from either LH_M_ or LH_M_-W^-^ (developing into red-eyed target males) in juvenile competition vials with 120 eggs from LH_M_-*bw* (developing into brown-eyed females and competitor males). After most flies had matured to adulthood (11.25 days, marking the end of the juvenile competition phase of the life cycle), a group of 5 red-eyed LH_M_ or LH_M_ -W^-^ target males was taken from each vial and placed into an fresh adult competition vial. To this vial we also added 11 brown-eyed LH_M_-*bw* competitor males and 16 brown-eyed LH_M_-*bw* females (these numbers made the density match the normal rearing conditions of LH_M_). The brown-eyed flies came from the same juvenile competition vials as the target males. After two days of mating competition, the 16 brown-eyed females were transferred to individual test tubes for egg-laying. Eighteen hours later, the females were discarded. Adult progeny from these test tubes (11.25 days old) were scored for red and brown eye color to determine paternity (red eyed progeny were from target sires and brown-eyed from competitors). In total, 500 infected and 500 uninfected males, having been mated to 2880 dams, were scored across 3 independent blocks.

We used different brown-eyed competitors (either LH_M_-*bw* or LH_M_-*bw*-W^-^) in the male and female fitness assay. This was done to avoid matings between individuals that would produce the CI phenotype.

Male fitness was analyzed using the same statistical approach as when analyzing female fitness. Male fitness was, however, quantified using two related, but different, measures; the proportion of offspring sired by the focal males per vial (taking the number of offspring from the competitor into account) and the number of offspring sired by the focal males. Both these measures have their benefits and drawbacks [38].

### Egg-to-adult survival of infected and uninfected eggs

Juvenile fitness (egg to adult survival) was measure for each sex and infection status by scoring the number of males and females that emerged from the 60 target eggs in each of the juvenile competition vials, used to produced males and females for the adult fitness assays.

Egg to adult survival was analyzed with a Generalized Linear Model (GLM) of the JMP 8.0 software package with binominal error terms, the logit link function and the number of individuals of the not-analyzed sex as a covariate. We analyzed each block separately and then applied a Consensus P-value test to combine inference across blocks [39].

### Total selection operating on *Wolbachia* cured females

To estimate the total selective effect on a female cured from *Wolbachia* infection, including both potential effects of fecundity and of CI, we deposited 30 eggs from either LH_M_ or LH_M_-W^-^ together with 150 eggs from LH_M_-*bw* into a juvenile competition vial. After 11.25 days we took 3 sets of 3 LH_M_ or 3 LH_M_-W^-^ females out of each of these vials and put them in an adult competition vial together with 13 LH_M_-*bw* competitor females and 16 LH_M_-*bw* males. Two days later the 3 target females were transferred to individual test tubes for egg laying, in which they spent 18 hours before they were discarded. The number offspring emerging from these vials were counted 11.25 days later and taken as a measure of fitness for cured females. The fitness of in total 540 infected and 540 uninfected females was scored, across 2 independent blocks. To analyze the total selection operation on cured females the same statistical approach was used as when analyzing female fitness above.

To estimate a confidence interval of the percent difference in fitness between infected and uninfected individuals (males, females and cured females in an environment with natural infection levels) we bootstrapped the data 100000 times. All bootstrap analyses were conducted using the program *Statistics101*.

## Results

### Cytoplasmic incompatibility

**Figure 1:**
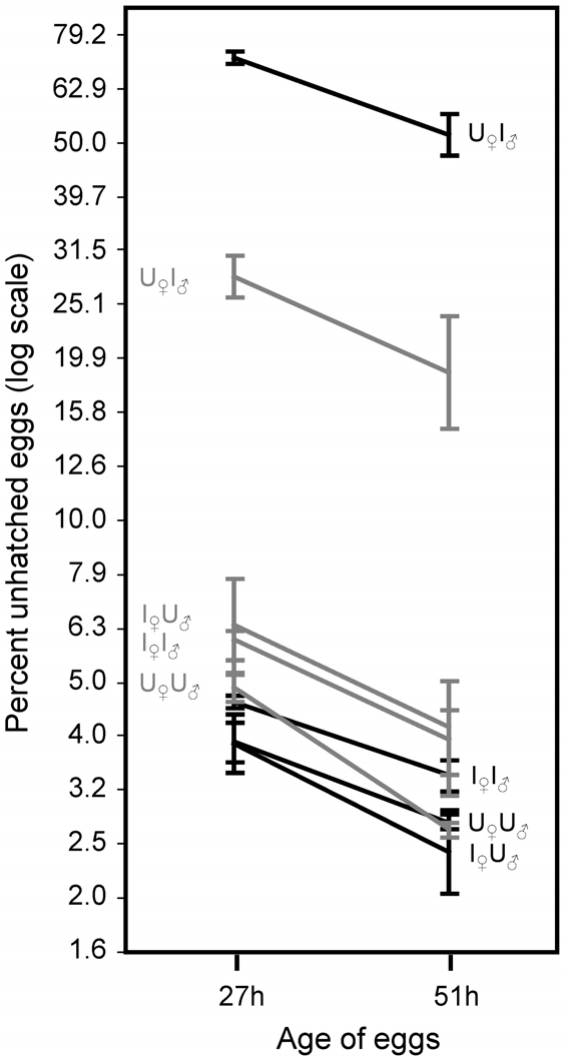
Cytoplasmic incompatibility: male age and embryonic development time. Proportion of unhatched eggs produced from all four combinations of infected and cured males and females. Males of two ages were used (black lines 1-day-old males, grey lines 3-day-old males) and eggs hatch was scored at two different times (first after 27 h and then again after 51 h). For each line there is a letter combination. The first letter correspond to the infection status (U = uninfected, I = infected) of the female (subscripted with ♀), the second letter refers to the infection status of the male (subscripted with ♂). Note that the Y-axis is log transformed.

There are several patterns that emerge from our analysis of CI, i.e., the hatchability of eggs in response to the infection status of sires and dams (and the age of the sire; Figure 1). The most obvious is that eggs from uninfected females sired by infected males have a highly reduced hatching rate compared to all the other crosses (Figure 1, Table 1), indicating substantial cytoplasmic incompatibility. Egg hatching rates from the three other crosses were more similar but contrast analyses show that the combination of infected females and infected males, on average, produced zygotes with lower hatchability compared to zygotes produced by uninfected females and uninfected males (t = 2.92 P = 0.0066) (Figure 1). Infected females and uninfected males produced intermediate hatch rates but did not significantly depart from the previous crosses (P>0.05 in all cases). As expected, the percent of unhatched eggs declined between the early (27 h) and late (51 h) sampling periods (Table 1, Figure 1). Although this decline was proportionately similar for all crosses, the actual decline was much larger for the CI cross which had substantially more unhatched eggs at the earlier sampling period (the decline was 14% for the CI cross 14% and 1.5–2% for all the other crosses). In the CI cross, older sires had lower rates of unhatched eggs, whereas this pattern was usually reversed among the compatible crosses (Figure 1 and Table 1).

**Table 1:** Analysis of variance of egg hatching rate.

Fixed Effects	*DF*	*F-ratio*	*P*
Cross	3	381.23	<0.0001
Male age	1	0.38	0.5409
Age of eggs	1	51.16	<0.0001
Cross × Male age	3	31.33	<0.0001
Cross × Age of eggs	3	0.21	0.8915
Male age × Age of eggs	1	0.90	0.3493
Cross × Male age × Age of eggs	3	0.25	0.8591

Random Effect	Var estimate	SE	Percent of Total
Block	0.000615	0.00110	7.45

‘Cross’ refers to the crosses combining infected and uninfected females in all four possible combinations. ‘Male age’ refers to one and three days old males. ‘Age of eggs’ refers to eggs scored at 27 h or 54 h after egg-laying.

### Population infection status

The proportion of low-hatch-rate broods (<80% hatch) was very similar between the CI-compatible and CI-incompatible crosses (21/994 for uninfected females mated to infected males and 20/996 for infected females mated to infected males), and not statistically different (Z = 0.16; P = 0.87). Using more stringent criteria (i.e. a higher threshold for the percent eggs that did not hatch) did not change this conclusion (data not shown). We used this difference in the proportion of low-hatch-rate broods to obtain a conservative estimate of the proportion of females losing the infection per generation. Bootstrapping our data 100,000 times, we estimated a 95% upper bound for any undetected excess in the proportion of low-hatch rate broods (in crosses that could involve matings between uninfected females and infected males) to be 0.91%, i.e., less than 1% of the females in the LH_M_ population were uninfected. This value can also be used as an upper bound of *μ*, the proportion of offspring that do not inherit *Wolbachia* from infected mothers.

### Egg-to-adult survival of infected and uninfected eggs

**Figure 2:**
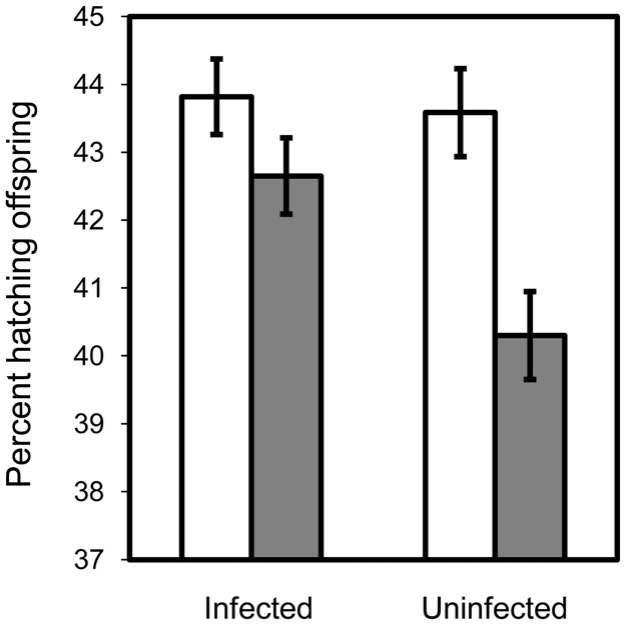
Effect of *Wolbachia* on egg-to-adult survival. Proportion of hatched eggs separated by sex for the infected (LH_M_) and uninfected population (LH_M_-W^-^).

We found no difference in the number of females emerging from our *Wolbachia*-infected and *Wolbachia*-uninfected populations in any block of our experiments (P>0.05 in all cases), nor did we find an effect when blocks were pooled with a consensus-P-value test (Z = 1.14, P = 0.256). However, a small excess of males emerged when the parents came from the infected population (Consensus P-value test, Z = 2.06, P = 0.040) (see Figure 2).

### *Wolbachia*'s influence on female and male fitness

We detected no effect on adult female fitness (number of eggs produced) when comparing infected females to females that had been cured from *Wolbachia* (mean number of offspring and S.E. for infected and cured females were 49.47 [0.36] and 49.77 [0.36]; F_1,117_ = 0.21, P = 0.6499, block variance estimate  = −0.193, SE = 0.057). The sample average fitness of infected females was 0.61% lower than uninfected female, but the 95% bootstrap confidence interval ranged from 3.21% lower fitness to 2.03% higher fitness.

We also could not detect any difference in fitness between infected and cured males. This was true both when fitness was measured as the proportion of offspring sired by the focal males (infected or cured) (mean_infected_ = 0.379, SE = 0.020 and mean_cured_ = 0.401, SE = 0.020; F_1,175_ = 1.83, P = 0.178, block variance estimate  = 0.001, SE = 0.001) and when fitness was measured as the number of offspring sired by the focal males (mean_infected_ = 17.33, SE = 1.15 and mean_cured_ = 18.00, SE = 1.15; F_1,175_ = 0.66, P = 0.417, block variance estimate  = 2.93, SE = 3.435). When using proportional data, the sample average fitness of infected males was 5.54% lower than that of cured males, (95% bootstrap confidence interval  = −14.10%, +1.71%). When using absolute numbers of offspring as the measure of male fitness the sample average reduction was 3.65% for infected males (95% bootstrap confidence interval  = −12.05%, 5.32%).

### Total selection operating on *Wolbachia* cured females

**Figure 3:**
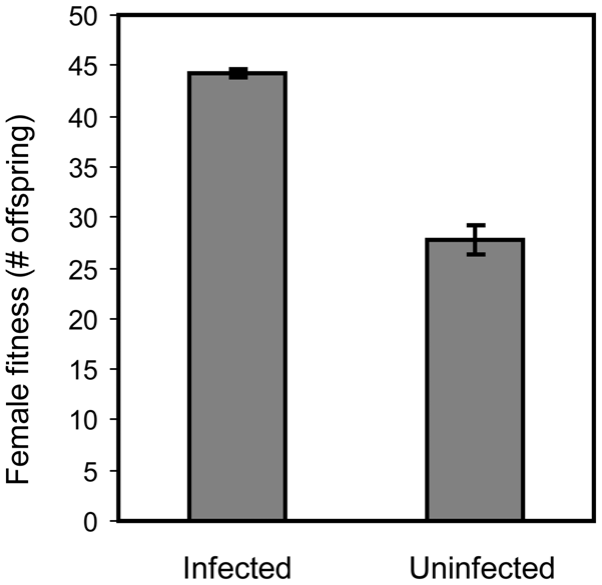
The cost of being cured of *Wolbachia*. Fitness of cured and infected females exposed to males from the base population (infected).

Females cured from *Wolbachia* had substantially reduced fitness compared to infected females (Figure 3, F_1,117_ = 447.42, P<0.0001, block variance estimate  = 0.007, SE = 0.62). The mean reduction in fitness for cured females was 37.18% (95% bootstrap confidence interval  = 34.35%, 39.94%).

## Discussion

The theory predicting the conditions under which CI-inducing *Wolbachia* can invade populations, and at what frequency *Wolbachia* is maintained (given successful invasion) has been well developed [12], [14], [15], [16]. To predict the fate of a *Wolbachia* infection requires knowledge of three parameters; *F*, the relative fecundity of infected to uninfected females, *μ*, the fraction of uninfected ova produced by infected females, and *H*, the relative hatch rate of eggs from incompatible (CI) to compatible crosses. In our population we estimated *F* to be 0.994 (with a 95% CI of 0.9682 to 1.0205) and an upper bound for *μ* to be 0.0091. We also measured the strength of CI to estimate *H*. Previous studies have shown that CI can depend on male age (e.g. [12]), and we therefore assayed CI using males of two age classes. Our results confirmed previous findings and showed that CI rapidly declines with male age. Estimating *H* from measures of CI, therefore, requires knowledge of both how CI and reproductive output relates to male age. To circumvent these problems we compared the fitness of cured and infected females experiencing the natural environment of this population, where virtually all individuals are infected (see results). By calculating the reduction in fitness of cured compared to infected females we got a more complete estimate of *H*, corresponding to 0.63 (lower and upper CI 0.60 and 0.66). This estimate of *H* also includes the difference in fecundity of cured and infected females, but given that this difference is very small in comparison to *H*, it does not substantially influence our conclusions.

The model referred to above has three equilibria, with reference to the proportion of infected individuals; *p_1_* = 0 (which can be stable or unstable), *p_2_* (which always is unstable when it exists) and *p_3_* (which can be stable or unstable) (for mathematical expressions of *p_2_* and *p_3_* see [16]). Two main questions of interest can be addressed using this model: i) can *Wolbachia* invade from an initially low frequency and ii) what, if any, is the stable infection equilibrium. The answer to the first question depends on the sign of F*(1-*μ*) [40]. If this quantity is larger than one, *p_1_* = 0 is unstable and *Wolbachia* can deterministically invade from a low initial frequency. If F*(1-*μ*) <1 invasion of *Wolbachia* relies on genetic drift, to raise the infection to a frequency above *p_2_*, from whence it will spread towards the upper stable equilibrium (given the conditions for *Wolbachia* are not so unfavorable that *p_1_* is globally stable, i.e. when 1-*H* ≤1-*F*). To predict if *Wolbachia* can invade the current population, given our estimated parameter values, we ran 10000 bootstraps to estimate the means and 95% CIs of F*(1-*μ*) and *p_2_*, setting *μ* = 0 if *μ* was estimated to be less than zero in any individual bootstrap. We also estimated the mean and 95% CI of *p_3_*, to quantify the expected equilibrium frequency of *Wolbachia*, given a successful invasion. These analyses show that it is unlikely, although possible, for *Wolbachia* to invade deterministically from an initially low frequency in this laboratory population (mean, lower and upper 95% CI of F*(1-*μ*) 0.992, 0.965, 1.019). Instead, *Wolbachia* would most likely have to spread through genetic drift, or by migration in the wild, to rise above the unstable *p_2_* (mean, lower and upper 95% CI of *p_2_* 2.10%, −5.1%, 9.5%). Given a successful invasion, the infection level is predicted to be very high at equilibrium (mean, lower and upper 95% CI of *p_3_* 99.6%, 97.9%, 100.0%), corresponding well to the current infection status in this population.

Our finding that *Wolbachia* has no effect on female fecundity in our population supports previous studies on *D. melanogaster* [21], [25], [27], [28]. However, despite a very large sample size we cannot completely rule out an effect, since the 95% CI of the relative fecundity of infected vs. uninfected females range from 0.9682 to 1.0205. The extreme values of this interval have little influence on the upper stable equilibrium (*p_3_*) [35], but in our population they cause a marked shift in the possibility for *Wolbachia* to invade, as they change F*(1-*μ*) from below to above one.

Assuming that our population was already infected with *Wolbachia* when it was brought into the lab more than 400 generations ago, we were surprised not to be able to detect a positive influence of *Wolbachia* on female fitness for two reasons. First, because theory predicts *Wolbachia* to be selected to maximize F*(1-*μ*) [16], [41], and second, because *Wolbachia* infecting a Californian population of *D. simulans* was recently shown to rapidly evolve to increase female fitness [35]. Our finding of no effect on male or female fitness does not, of course, preclude the possibility that the *Wolbachia* strain infecting our population has a positive effect outside the laboratory, where flies are expected to encounter a more variable environment. Recent studies have, for example, found that *Wolbachia* can have positive effects on both resistance to viruses [8] as well as on iron homeostasis [9]. Nonetheless, *Wolbachia* seems to have evolved the mutually beneficial phenotype of not harming the reproductive output of females that carry it, assuming this is not the result of the nuclear genome evolving to neutralize any negative effects of *Wolbachia*.

Earlier studies have shown that the nuclear genome is constrained with respect to optimizing the different phenotypes that maximize fitness of males and females (reviewed in [42]). *Wolbachia* is, however, unconstrained with respect to mutations that benefit female while harming male phenotypes, since *Wolbachia* is exclusively propagated across multiple generations via the matriline. This asymmetry motivated us to also compare the fitness of infected and cured males. Due to higher phenotypic variation in male fitness our estimate of *Wolbachia's* influence on male fitness is less accurate than the one that we obtained for female fitness. The estimated fitness of infected males was 3.65% lower than that of uninfected males, but the 95% CI of this difference overlapped zero (−12.05%, 5.32%). The point estimates of *Wolbachia's* effect on fitness thus suggests that *Wolbachia*, if anything, has a negative effect on both sexes, with this effect possibly being more pronounced in males. Our study cannot rule out small fitness effects (positive or negative) of carrying *Wolbachia* on both males and females.

When there is sibling competition, theory predicts that *Wolbachia* should be selected to kill or reduce the competitive ability of male offspring, thereby freeing-up resources for daughters who later transmit *Wolbachia*. Sibling competition is not strong in our population, since larvae intermixed from 16 females compete over resources, and therefore we did not expect *Wolbachia* to evolve a strong negative effect on juvenile males (unless this phenotype was a relict of past selection in wild populations). We were, however, surprised to find that *Wolbachia* had a significantly positive influence on male but not on female juvenile survival (see figure 2). We can only speculate on why this is the case. Both males and females have coevolved with the persistent infection of *Wolbachia* for long periods of time. It may be that some component of male function – but not female function - has become partially dependent on the presence of *Wolbachia* in the cytosol. However, the positive effect on male juvenile survival was only marginally significant (P = 0.04), so more data is needed to fully evaluate this result.

Our finding that embryos from parents that both were infected with *Wolbachia* had better hatch rate than embryos from parents that did not carry *Wolbachia*, was also surprising. This result may be due to prolonged coevolution of males and females with their endosymbiont, as embryos from infected parents carry *Wolbachia* and do not suffer increased male juvenile survival. This explanation is not fully satisfactory, since embryos from infected mothers and cured fathers produced embryos with intermediate survival, despite the fact that all of these male embryos carried *Wolbachia*.

In summary, our data show that *Wolbachia* is maintained within our large outbred laboratory population (once it was established), because of the low fraction of uninfected ova from infected females (*μ*) in combination with a substantial level of CI. *Wolbachia* prevalence is sufficiently high, and CI sufficiently strong, that there is very strong selection (*s* = −0.37) on the nuclear genome against females losing their infection. Likewise, selection currently acts on the nuclear genome to minimize *μ*. These factors indicate that the *Wolbachia* infection is likely to persist indefinitely in the LH_M_ population. It has been suggested that *Wolbachia* needs to have a positive effect on female fitness to be maintained in natural populations of *D. melanogaster* [25], [26], [43]. However this is clearly not the case for our laboratory population, where no such benefit can be found, despite persistent infection.
